# Functional Popliteal Artery Entrapment Syndrome: Poorly Understood and Frequently Missed? A Review of Clinical Features, Appropriate Investigations, and Treatment Options

**DOI:** 10.1155/2014/105953

**Published:** 2014-09-07

**Authors:** Matthew Hislop, Dominic Kennedy, Brendan Cramp, Sanjay Dhupelia

**Affiliations:** ^1^Brisbane Sports and Exercise Medicine Specialists Clinic, 87 Riding Road, Brisbane, QLD 4171, Australia; ^2^Queensland X-Ray, Greenslopes Private Hospital, Brisbane, QLD 4120, Australia

## Abstract

Functional popliteal artery entrapment syndrome (PAES) is an important and possibly underrecognized cause of exertional leg pain (ELP). As it is poorly understood, it is at risk of misdiagnosis and mismanagement. The features indicative of PAES are outlined, as it can share features with other causes of ELP. Investigating functional PAES is also fraught with potential problems and if it is performed incorrectly, it can result in false negative and false positive findings. A review of the current vascular investigations is provided, highlighting some of the limitations standard tests have in determining functional PAES. Once a clinical suspicion for PAES is satisfied, it is necessary to further distinguish the subcategories of anatomical and functional entrapment and the group of asymptomatic occluders. When definitive entrapment is confirmed, it is important to identify the level of entrapment so that precise intervention can be performed. Treatment strategies for functional PAES are discussed, including the possibility of a new, less invasive intervention of guided Botulinum toxin injection at the level of entrapment as an alternative to vascular surgery.

## 1. Introduction

Functional popliteal artery entrapment syndrome (PAES) is an important and possibly underrecognized cause of exertional leg pain. It shares many features with other causes of exertional leg pain, and more than one condition can be present at once, confusing the clinical picture. An understanding of the typical presenting features of the common causes of exertional leg pain is essential, allowing the clinician to determine those suggestive of PAES and requiring further investigation.

Investigating functional PAES is fraught with potential problems and, if performed incorrectly, can result in false negative and false positive findings. The authors believe that currently accepted vascular investigations such as ankle-brachial indices and Doppler ultrasound performed at rest are not accurate in investigating functional PAES. Rather, a review of the literature would suggest that investigations such as provocative Doppler ultrasound and MRI angiography are performed as soon as possible after reproducing symptoms to “capture” the occlusion while it is occurring.

Once the diagnosis of functional PAES is confirmed, there are a number of treatment strategies available. Until recently, definitive intervention was only available in the form of vascular surgery with variable myotomies and releases. We provide information on a Pilot study suggesting a new less invasive intervention of guided botulinum toxin injection to the level of entrapment, as an alternative to surgical intervention.

## 2. Clinical Features and Differential Diagnosis of Exertional Leg Pain

PAES shares many clinical features with other causes of exertional leg pain, most of which are thought to be more common [1]. Adding to the complexity, more than one cause of exertional leg pain may be present in an individual patient at any one time. Chronic exertional compartment syndrome (CECS) in particular has many of the same features of PAES and the two conditions can be confused [2, 3]. Also, it is not possible to distinguish between anatomical and functional PAES on clinical symptoms alone. A detailed history and examination provide very useful information and help determine whether further investigation is warranted. An outline of the more common differential diagnoses of exertional leg pain and their features follows and a summary is provided in Table 1.

### 2.1. Medial Tibial Stress Syndrome (MTSS)

This condition is thought to represent the most common cause of exertional leg pain [4] with an incidence between 13 and 42% [5]. Patients describe typically bilateral leg pain along a strip of the distal third of the posteromedial tibial border that comes on early with exercise and may warm up but usually will ache for some time after stopping [6]. The pain is also impact related [7]. Examination at rest will usually reveal palpable tenderness along the distal third of the posteromedial tibial border and pain with hopping [6]. Some studies suggest a possible predisposition of MTSS in females [8].

### 2.2. Stress Fractures

While stress fractures are not common in the nonexercising population, they are thought to represent 0.7–20% of sports medicine clinic presentations [6]. Patients typically complain of pain that is focal and palpable (unless the site is covered by a significant muscle layer where symptoms can be vague and nondescript). With lower limb stress fractures, pain often presents early with impact related exercise and is worse during the landing phase of running [7]. Stress fractures are typically unilateral and can affect any bone in the lower limbs, although the tibia is most common [1, 9]. Female athletes are thought to be at greater risk of developing stress fractures than males [8] and Bennell et al. suggest that menstrual disturbances, caloric restriction, lower bone density, muscle weakness, and leg length differences are important risk factors for stress fracture [10].

### 2.3. CECS

CECS is thought to have an incidence between 27 and 33% [5]. Patients by definition are pain-free at rest and develop a steadily worsening pain with exertion [11]. The pain will gradually build up over 10–20 minutes and is not rapid in onset (unlike some cases of PAES). The pain is typically isolated to one or more compartments, most commonly anterior and deep posterior, with the least most common being the superficial posterior compartment (where the majority of PAES pain is described) [5, 9]. Patients describe feelings of “hardness” in their affected compartment. The pain will typically dissipate slowly over minutes to hours but will return quickly if the patient attempts to return to exercise again. Fascial defects are more common but not diagnostic [1, 5]. The leg pain is overwhelmingly bilateral (although unilateral cases can present) and there is no difference in predisposition according to the sex of the individual [7, 11].

### 2.4. PAES

PAES can be further divided into two groups, anatomical and functional. In the case of anatomical PAES, there is a clearly defined anatomical lesion that directly leads to entrapment and subsequent occlusion of the popliteal artery. The second and larger subgroup of anatomically “normal” or functional PAES is particularly poorly understood [2]. In functional PAES, there is evidence of popliteal artery occlusion and subsequent claudicant symptoms, but no defined lesion can be found that directly causes the occlusion.

Anatomical and functional PAES most likely present in a similar fashion. Patients with anatomical PAES are thought to be older and more sedentary while functional PAES patients are thought to be younger, more commonly female, and more active [3]. Baltopoulos et al. suggest that functional PAES may be bilateral in 25–76% of cases [12]. The true incidence of functional PAES is unknown, and it is described as being rare [8, 13, 14]. However, it is possible that this condition is underreported [15] and the incidence may be greater than previously recognized. The pain is often vague and poorly localized. It occurs most commonly in the “back of the calf” but it can be anterior and lateral especially if the anterior tibial artery is involved [2, 16]. Patients can have pain with minimal exertion or provocative leg positioning and occasionally at rest. Symptoms such as coldness and paraesthesia have also been reported [17]. Running or walking on an incline can occasionally bring on symptoms [3]. The pain follows a claudicant pattern similar to CECS but unlike CECS will resolve more quickly on cessation of exercise [18]. However, an ache may persist for hours. Provocative manoeuvres may result in the development of a reduced peripheral pulses and/or a popliteal bruit on auscultation of the popliteal fossa [7] although this may also be possible in the “asymptomatic occluder” group as outlined later.

## 3. Examination Findings in Functional PAES

When examining patients with suspected functional PAES, it is important to diagnose and/or exclude other causes of exertional leg pain. Examination includes palpation and percussion over the lower limb to look for signs of bone stress, be it focal at the site of a stress fracture or along a strip of the distal posteromedial tibial border in the case of MTSS. Some authors suggest that PAES must have a combination of pain with hopping, in conjunction with the development of ischaemic signs like pallor, coldness, and reactive hyperaemia [18]. In our experience, pain with hopping more likely indicates some form of bone stress, and the development of physical signs of ischaemia in younger individuals with suspected functional PAES is extremely uncommon.

### 3.1. Clinical Vascular Provocation Test

A validated clinical provocation test for PAES has not been described in the literature. Some authors describe physical examination as unreliable [7] and rely on clinical suspicion as justification for progressing to vascular investigations. Some do not describe their clinical assessment [2], while others attempt to provoke symptoms by encouraging the patient to hop or climb stairs, followed by a cursory physical examination [18]. Most will assess resting peripheral pulses [12, 18] which should be examined at true rest. The popliteal fossa should be auscultated to determine the presence of a resting bruit. In general, bruits are not audible until an artery is approximately 50% occluded [19]. The sound increases in pitch as the lumen becomes more narrowed to a critical size. Thereafter, the sound may no longer be detectable as the volume of blood flow becomes greatly reduced [19]. If pulses are reduced or a bruit is present at rest, than this would indicate an underlying vascular malformation or significant luminal narrowing (such as in advanced atherosclerosis) and further radiological imaging is indicated.

We have developed a simple clinical test that can be performed in the consulting rooms. After assessing for a popliteal fossa bruit and examining peripheral pulses at rest, the patient is required to perform 15–20 single leg eccentric heel drops off the edge of a step, while asking about any developing or worsening leg pain. Immediately after the test is performed, the popliteal fossa is again auscultated for a bruit, and peripheral pulses are examined for any reduction. In our experience, it is worth auscultating for a number of minutes after exertion, as more significant cases (with complete occlusion) may initially have no bruit, or at least delayed onset of a bruit as flow reestablishes.

We believe that if patients do not develop pain or discomfort with this test, or if a bruit and/or pulse reduction is not evident, then it is unlikely that the patient is suffering from PAES. Unfortunately, the development of pain/bruit/pulse reduction does not mean the patient has PAES, as it is possible that they may fall into the asymptomatic occluder group. Provided that the patient has clinical features suggestive of PAES, a positive result warrants further vascular investigation.

## 4. Investigations in Functional PAES

Despite PAES being a well-defined condition, no clear-cut consensus regarding the diagnostic work-up of these patients exists [20]. The general population can be divided into four groups, including asymptomatic nonoccluders, who presumably will not present for assessment or investigation. Therefore, the three symptomatic groups that investigation must distinguish are

1. anatomical PAES,
2. asymptomatic occluder (i.e., exertional leg pain due to another cause, but in whom the artery can incidentally occlude),
3. symptomatic occluder, that is, functional PAES.

### 4.1. Ankle Brachial Indices (ABIs)

The ABI is calculated by dividing the systolic blood pressure at the ankle by that at the arm. Measures below 0.8 suggest at least moderate peripheral vascular disease. Some authors recommend the use of ankle brachial indices in standard work-up for functional PAES [3]. However, as occlusion in functional PAES occurs during exertion, testing after exercise is likely to result in a false negative result [20]. There are also difficulties in obtaining ABI measurements during exercise [18] and Pillai suggests that ABIs during forceful plantarflexion are difficult to interpret and not as helpful when assessing graded compression of a patent artery [17]. We agree that ABIs are unreliable for investigating functional PAES.

### 4.2. Compartment Pressure Testing

Some authors will routinely perform compartment pressure studies in addition to vascular studies in the work-up of suspected PAES [3]. This seems to be excessive and our review of the literature could find no compelling reason as to why this should be mandatory. For this reason we do not recommend routine compartment pressure studies, unless symptoms and history are strongly suggestive of possible concomitant CECS.

### 4.3. Doppler Ultrasound

Doppler ultrasound provides a relatively cheap, noninvasive, and accessible procedure to assess flow through the popliteal artery, and it is generally recommended that this be the first line investigation for PAES [20, 21]. Despite this, a review of the literature shows no definite consensus on how to perform Doppler ultrasound in investigating PAES [20]. Some authors suggest hand held Doppler units to complement clinical assessment in the rooms. This assesses loss of the posterior tibial artery distal pulse during sustained passive dorsiflexion and plantarflexion of the foot [12]. Doppler ultrasound is user dependent, which can affect reliability. Also, there is a risk of overcalling entrapment with movement of the artery, muscles, or probe during exercise giving the illusion of occlusion [21], but technique variations can usually overcome this.

#### 4.3.1. Provocative Doppler Protocols

Many authors researching PAES suggest that the most important feature in its diagnosis is the reproduction of symptoms with the help of provocative manoeuvres and verification by duplex ultrasonography [17, 18, 21]. Typically, investigations at rest will show a patent popliteal artery with normal distal pulses. In functional PAES, occlusion will often only occur during exertion and can resolve almost immediately. This can give the false impression of no pathology if the assessment is delayed. Even the 30–60 seconds taken from getting off a treadmill to an examination bed and applying the ultrasound probe can be enough for arterial occlusion to cease. Because occlusion can resolve very quickly, immediate assessment after reproduction of symptoms is essential [18]. The position of most occlusions occurs when the patient has their knee extended, and they hold active plantarflexion. It is not possible to hold this position for sustained periods during a MRI due to discomfort and fatigue [21]. Ultrasound is a useful modality, as it allows real time assessment whilst some form of provocation to reproduce symptoms is performed. Despite the agreement that provocation is necessary, a clear and detailed description of the protocols to do this is generally not provided [20]. Our protocols are included below for completeness.

We perform a resting anatomical study first, with the patient in the supine position. We record waveforms and velocities of the peripheral arteries as well as assess for evidence of intimal thickening or fixed arterial disease. The anatomy of the popliteal fossa is assessed, looking particularly for any popliteal artery or soft tissue structure variations or whether the anterior tibial artery has a high bifurcation.

### 4.4. Provocation

Hoffman et al. [22] found that the force of plantarflexion required to occlude the popliteal artery during provocative positioning is important, with the majority of asymptomatic occluders occluding the popliteal artery with sustained holding of ≥70% of maximal plantarflexion force [22]. For this reason, we use a graded ultrasound protocol progressing through

**Figure 1:**
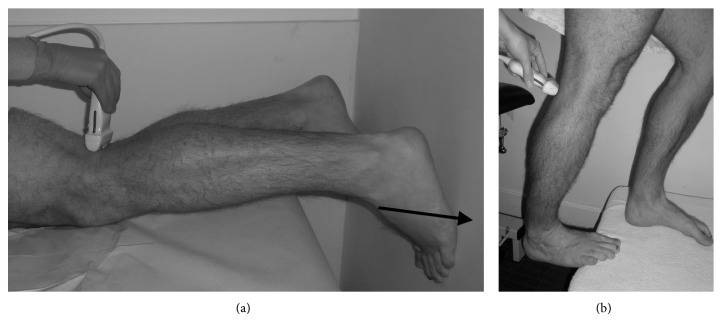
(a) Patient prone and pushing against a wall (in the direction of the arrow) at 25% maximum plantarflexion force. (b) Patient erect and plantarflexing against full body weight.

1. prone patient holding a position of provocation (knee extension and plantarflexion) against no load,
2. patient is prone and pushing against approximately 25% of maximal plantarflexion force (Figure 1(a)),
3. dynamic loading by pushing against 50–100% of maximal plantarflexion force (Figure 1(b)).

Symptomatic patients who occlude in the first 2 categories are presumed to represent more severe cases of functional PAES. If the initial 2 assessments are negative, the patient is assessed in an erect weight-bearing position while cycling through range of motion. This is thought to represent somewhere between 50 and 100% maximal plantarflexion force and may more reliably represent what is occurring functionally during activity. If the erect assessment is negative, the patient may attempt similar exertion that elucidates symptoms in a normal training experience, until they are symptomatic.

At this point, we again assess the patient in an erect position as previously described. We will use cine loops to demonstrate the popliteal artery from resting patency through compression to occlusion and mark the site of compression on skin for MRI correlation. We feel that Doppler ultrasound does not adequately demonstrate the anatomy to rule out anatomical PAES and confirmation of occlusion in a symptomatic patient means the patient may be in either the anatomical or functional PAES groups.

### 4.5. MRI and MRI Angiography

MRI is a valuable noninvasive modality that allows optimal visualization of the popliteal artery as well as the surrounding structures [23, 24]. MRI can also distinguish intrinsic vascular disease from extrinsic compression. MRI can demonstrate a variety of findings including abnormal lateralized insertion of the medial head of gastrocnemius, medial displacement, and occlusion of the popliteal artery in the popliteal fossa and fat tissue filling the normal location of the medial head of gastrocnemius [23, 25]. In this way, anatomical PAES can be distinguished from functional PAES and the muscles and anatomical boundaries contributing can be accurately delineated [3]. This in turn can direct injection therapy (as discussed in Section 5) or the site of surgical intervention.

MRI angiography can demonstrate the level of occlusion, but limitations include the development of movement artifact with forceful plantarflexion positions and inability to hold such a position for prolonged periods [17]. This is a significant limitation as for reasons outlined earlier; investigating for occlusion in functional PAES should occur during provocation. Most papers reviewed do not describe provocative manoeuvres with MRI angiography [23–25] while one suggests holding sustained forceful plantarflexion continuously for 20–29 seconds whilst the angiogram is performed [26]. Again for completeness we have provided our protocols below.

After positive ultrasound studies, a fiducial marker is placed on the patient's skin where the popliteal artery is being occluded to help correlate between the ultrasound and MRI site of occlusion. T1 weighted axial and coronal images are acquired to demonstrate the medial head and lateral heads of gastrocnemius, popliteus, and plantaris muscles and their alignment and associations with the femoral condyles and popliteal arteries and nerves. These images are acquired with the patient at rest.

**Figure 2:**
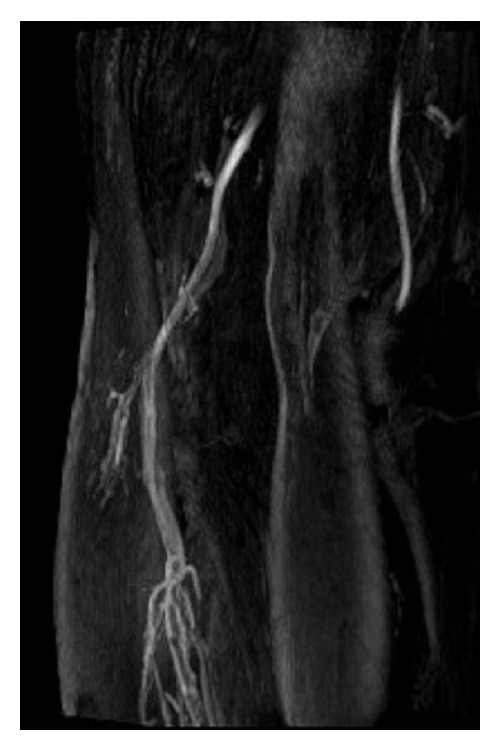
MRI angiogram of the popliteal fossa showing complete occlusion of the popliteal artery in the left leg.

Following these static images, the patient is instructed to dorsiflex and plantarflex their feet whilst acquiring T2 weighted 2D steady state images axially across the popliteal region. Before the final contrast MRI angiogram is performed, the patient is instructed to alternate a neutral ankle position with maximal plantarflexion force until they stimulate the pain that they usually experience (rather than a single sustained forceful contraction). Once they experience this pain, they keep their ankles in plantarflexion whilst we inject the contrast and perform the angiogram (Figure 2). One of the disadvantages of this technique is that there is an approximate 30-second delay before the contrast arrives at the popliteal artery and the angiogram can be performed and the artery may re-establish flow during this time. Patients quite often are in pain and or exhausted during this last series and may shake because of this.

## 5. Treatment

**Figure 3:**
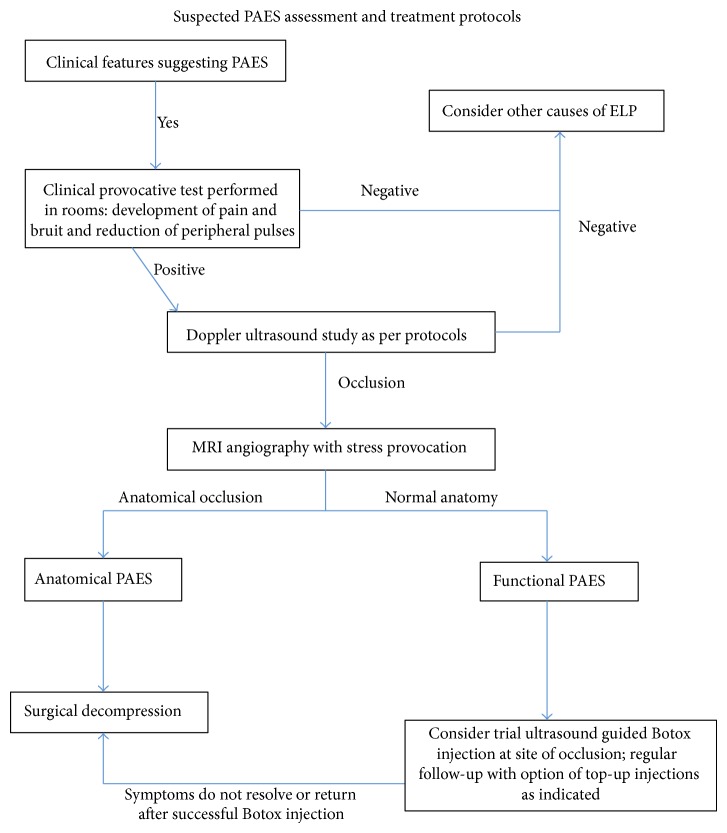
Assessment and treatment protocols for suspected PAES.

Based on our review of the literature, Figure 3 represents a decision-making flowchart in the assessment, work-up, and treatment of suspected PAES. Once confirmed, treatment depends on the type of PAES identified.

### 5.1. Anatomical PAES

Given the described rapid progression of arterial injury, it is recommended that anatomical PAES patients undergo surgery to remove the site of entrapment [17]. This typically involves exploration, limited fasciotomy, myotomy to varying degrees, and possible excision of occlusive fibrous bands [2, 17]. Results of popliteal fossa exploration, bypass, or muscle detachment, or a combination of these, and fossa decompression are generally good. Most series report a small number of patients, but >90% appear to return to activities in sports ≤ 3 months with resolution of all previous symptoms [17].

### 5.2. Functional PAES

The pathogenesis and progression of functionalPAES are uncertain, but it may be that these patients develop arterial injury more gradually with onset of more significant symptoms later in life [12]. Surgery has been recommended in functional occluders with significant repetitive and typical symptoms [17]. Turnipseed recommends resection of plantaris muscle and the crural band of soleus fascia that forms the outlet of the popliteal fossa as he feels that this fascial band is the fulcrum against which the neurovascular bundle is compressed in functional PAES [3].

However the site and amount of muscle necessary to be removed to prevent further occlusion is not always obvious. It is possible that a large segment of muscle will require excision. The popliteal artery will need exploration and there are increased risks of postoperative complications such as seroma (4.6%) and infection (2%) [2]. Also surgery for functional PAES does not seem to be as successful as that for anatomical PAES, with reports suggesting only 77% of patients after surgery reporting complete resolution of symptoms [14]. For this reason, a less invasive treatment option is desirable.

### 5.3. Guided Botulinum Toxin (Botox BTX-A) Injection Therapy

The use of botulinum injections for paralysis of muscles to manage medical conditions is well established. There are several descriptions of the use of botulinum in the treatment of muscle spasticity, particularly in cerebral palsy patients [27–29], and piriformis injection of BTX-A has been used successfully to treat sacral plexus and proximal sciatic nerve compression [30]. Bilici et al. described the use of BTX-A injection into a crus of the hemidiaphragm to treat renal artery stenosis [31].

The proposed mechanisms of action for intramuscular periarterial botulinum therapy for PAES are

1. paralysis of the muscular slip of muscle responsible for the dynamic arterial occlusion,
2. localised muscle atrophy caused by the botulinum which may increase space for the vessel and would explain the prolonged effect of botulinum on this condition beyond the expected therapeutic effect of the medication,
3. possible arterial smooth muscle relaxation of the popliteal artery resulting in vasodilatation.

Unfortunately, to date, there is no published data on the efficacy of botulinum injection in the management of functional PAES. We have commenced a pilot study using intramuscular periarterial injection of BTX-A to treat functional PAES with promising initial results. We hope to publish the outcomes as our cohort size increases, but at present this remains an unproven intervention.

## 6. Summary

Functional PAES is a condition that is possibly underrecognized and, if left untreated, can result in progressive arterial damage and the risk of developing lower limb ischaemia. It shares many features with other causes of exertional leg pain (especially chronic exertional compartment syndrome) and may coexist with one or more of these. A suggestive clinical history includes features of pain aggravated by exercise, but also possibly at rest with positions of knee extension and plantarflexion. The pain will typically resolve quickly once provocative manoeuvres are ceased, although an ache may persist for hours. Anatomical and functional PAES cannot be distinguished on clinical features alone, and possibly over half of the “normal” population can demonstrate some arterial occlusion with provocative manoeuvres. For this reason, specialized vascular investigations are indicated, particularly a Doppler ultrasound protocol performed at rest and during provocation and immediately after, which can demonstrate real time arterial occlusion and the level it is occurring at. Once occlusion is demonstrated, MRI can demonstrate the definitive anatomy of the popliteal fossa, whether anatomical PAES exists, and the site and extent of functional entrapment. From here, the best treatment can be provided, with consideration of guided Botox injection for functional PAES as a potential new intervention, or progression to surgical intervention.

## Supplementary Material

A video loop of Doppler Ultrasound has been provided as supplementary material. This shows a cross section of the popliteal fossa, and demonstrates a patent popliteal artery at rest. The patient was then instructed to forcibly plantarflex their ankle, and the flow in the artery is seen to stop as occlusion occurs.

## Figures and Tables

**Table 1:** Differential diagnosis and clinical features of exertional leg pain.

Condition	Incidence	Male/female preponderance	Unilateral/bilateral tendency	Site of pain	Pain present at rest	Pattern of pain
MTSS	13–42%	Possibly female	Bilateral	Posteromedial tibial border	Yes (on palpation)	Pain with activity can warm up and returns on cessation

Stress fracture	Unknown (0.7–20% exercising population)	Possibly female	Unilateral	Variable depending on site of stress fracture	Yes (on palpation)	Pain with impact activity

CECS	27–33%	Nil	Bilateral	Typically anterior and/or deep posterior compartments	No	Crescendo-decrescendo pattern: pain can last for minutes to hours on cessation

PAES (anatomical)	0.6–3.5% (rare)	Possibly male	Possibly unilateral	Typically superficial posterior compartment	Can be at rest (positional)	Crescendo-decrescendo pattern: pain can last for seconds to minutes on cessation

PAES (functional)	Unknown (possibly common and underrecognized)	Possibly female	Likely bilateral	Typically superficial posterior compartment	Can be at rest (positional)	Crescendo-decrescendo pattern: pain can last for seconds to minutes on cessation

## References

[1] Blackman P. G. (2000). A review of chronic exertional compartment syndrome in the lower leg. *Medicine & Science in Sports & Exercise*.

[2] Turnipseed W. D. (2009). Functional popliteal artery entrapment syndrome: a poorly understood and often missed diagnosis that is frequently mistreated. *Journal of Vascular Surgery*.

[3] Turnipseed W. D. (2012). Popliteal entrapment in runners. *Clinics in Sports Medicine*.

[4] Thacker S. B., Gilchrist J., Stroup D. F., Kimsey C. D. (2002). The prevention of shin splints in sports: a systematic review of literature. *Medicine and Science in Sports and Exercise*.

[5] George C. A., Hutchinson M. R. (2012). Chronic exertional compartment syndrome. *Clinics in Sports Medicine*.

[6] Reshef N., Guelich D. R. (2012). Medial tibial stress syndrome. *Clinics in Sports Medicine*.

[7] Pham T. T., Kapur R., Harwood M. I. (2007). Exertional leg pain: teasing out arterial entrapments. *Current Sports Medicine Reports*.

[8] Reinking M. (2007). Exercise Related Leg Pain (ERLP): a review of the literature. *North American Journal of Sports Physical Therapy*.

[9] Hislop M., Tierney P., Murray P., O'Brien M., Mahony N. (2003). Chronic exertional compartment syndrome: the controversial “fifth” compartment of the leg. *The American Journal of Sports Medicine*.

[10] Bennell K., Matheson G., Meeuwisse W., Brukner P. (1999). Risk factors for stress fractures. *Sports Medicine*.

[11] Hislop M., Batt M. E. (2011). Chronic exertional compartment syndrome testing: a minimalist approach. *British Journal of Sports Medicine*.

[12] Baltopoulos P., Filippou D. K., Sigala F. (2004). Popliteal artery entrapment syndrome: anatomic or functional syndrome?. *Clinical Journal of Sport Medicine*.

[13] Love J. W., Whelan T. J. (1965). Popliteal artery entrapment syndrome. *The American Journal of Surgery*.

[14] Sinha S., Houghton J., Holt P. J., Thompson M. M., Loftus I. M., Hinchliffe R. J. (2012). Popliteal entrapment syndrome. *Journal of Vascular Surgery*.

[15] Levien L. J., Veller M. G. (1999). Popliteal artery entrapment syndrome: more common than previously recognized. *Journal of Vascular Surgery*.

[16] Mavili E., Dönmez H., Kahriman G., Özaşlamaci A., Özcan N., Taşdemir K. (2011). Popliteal artery branching patterns detected by digital subtraction angiography. *Diagnostic and Interventional Radiology*.

[17] Pillai J. (2008). A current interpretation of popliteal vascular entrapment. *Journal of Vascular Surgery*.

[18] Lane R., Nguyen T., Cuzzilla M., Oomens D., Mohabbat W., Hazelton S. (2012). Functional popliteal entrapment syndrome in the sportsperson. *European Journal of Vascular and Endovascular Surgery*.

[19] Hill R. D., Smith R., Walker H. K., Hall W.D., Hurst J. W. (1990). Examination of the extremities: pulses, bruits, and phlebitis. *Clinical Methods: The History, Physical, and Laboratory Examinations*.

[20] Altinatis U., Helgstrand U., Hansen M. (2013). Popliteal artery entrapment syndrome: ultrasound imaging, intra-operative findings and clinical outcome. *Vascular and Endovascular Surgery*.

[21] Erdoes L. S., Devine J. J., Bernhard V. M., Baker M. R., Berman S. S., Hunter G. C. (1994). Popliteal vascular compression in a normal population. *Journal of Vascular Surgery*.

[22] Hoffmann U., Vetter J., Rainoni L., Leu A. J., Bollinger A. (1997). Popliteal artery compression and force of active plantar flexion in young healthy volunteers. *Journal of Vascular Surgery*.

[23] Atilla S., Ilgit E. T., Akpek S., Yücel C., Turgut Tali E., Iik S. (1998). MR imaging and MR angiography in popliteal artery entrapment syndrome. *European Radiology*.

[24] Lambert A. W., Wilkins D. C. (1999). Popliteal artery entrapment syndrome. *The British Journal of Surgery*.

[25] Özkan U., Oğuzkurt L., Tercan F. (2008). MRI and DSA findings in popliteal artery entrapment syndrome. *Diagnostic and Interventional Radiology*.

[26] Di Cesare E., Marsili L., Marino G. (1994). Stress MR imaging for evaluation of popliteal artery entrapment. *Journal of Magnetic Resonance Imaging*.

[27] Corry I. S., Cosgrove A. P., Walsh E. G., McClean D., Graham H. K. (1997). Botulinum toxin A in the hemiplegic upper limb: a double blind trial. *Developmental Medicine and Child Neurology*.

[28] Fehlings D. (2005). The use of botulinum toxin in paediatric hypertonia. *Paediatrics and Child Health*.

[29] Koman L. A., Mooney J. F., Smith B. P., Goodman A., Mulvaney T. (1994). Management of spasticity cerebral palsy with botulinum—a toxin: report of a preliminary, randomized, double-blind trial. *Journal of Pediatric Orthopaedics*.

[30] Yoon S. J., Ho J., Kang H. Y. (2007). Low-dose botulinum toxin type A for the treatment of refractory piriformis syndrome. *Pharmacotherapy*.

[31] Bilici A., Karcaaltincaba M., Ilica A. T., Bukte Y., Senol A. (2007). Treatment of hypertension from renal artery entrapment by percutaneous CT-guided botulinum toxin injection into diaphragmatic crus as alternative to surgery and stenting. *The American Journal of Roentgenology*.

